# Patient-reported patterns of follow-up care in the ‘Aftercare in Blood Cancer Survivors’ (ABC) study

**DOI:** 10.1007/s00432-023-04889-7

**Published:** 2023-06-08

**Authors:** Julia Baum, Hildegard Lax, Nils Lehmann, Anja Merkel-Jens, Dietrich W. Beelen, Karl-Heinz Jöckel, Ulrich Dührsen

**Affiliations:** 1 Klinik für Hämatologie, Universitätsklinikum Essen, Universität Duisburg-Essen, Hufelandstraße 55, 45147 Essen, Germany; 2 Institut für Medizinische Informatik, Biometrie und Epidemiologie, Universität Duisburg-Essen, Essen, Germany; 3 Klinik für Knochenmarktransplantation, Universitätsklinikum Essen, Universität Duisburg-Essen, Essen, Germany

**Keywords:** Allogeneic transplantation, Blood cancer, Follow-up care, Leukemia, Lymphoma, Myeloproliferative neoplasm, Quality of life

## Abstract

**Background:**

Follow-up care provides long-term support for cancer survivors. Little is known about follow-up care in hematologic malignancies.

**Methods:**

Our questionnaire-based study included blood cancer survivors diagnosed at the University Hospital of Essen before 2010, with a ≥ 3-year interval since the last intense treatment. The primary goal of the retrospective study was the identification and characterization of follow-up institutions.

**Results:**

Of 2386 survivors meeting the inclusion criteria, 1551 (65.0%) consented to participate, with a follow-up duration > 10 years in 731. The university hospital provided care for 1045 participants (67.4%), non-university oncologists for 231 (14.9%), and non-oncological internists or general practitioners for 203 (13.1%). Seventy-two participants (4.6%) abstained from follow-up care. The disease spectrum differed among follow-up institutions (*p < *0.0001). While allogeneic transplant recipients clustered at the university hospital, survivors with monoclonal gammopathy, multiple myeloma, myeloproliferative disorders, or indolent lymphomas were often seen by non-university oncologists, and survivors with a history of aggressive lymphoma or acute leukemia by non-oncological internists or general practitioners. Follow-up intervals mirrored published recommendations. Follow-up visits were dominated by conversations, physical examination, and blood tests. Imaging was more often performed outside than inside the university hospital. Satisfaction with follow-up care was high, and quality of life was similar in all follow-up institutions. A need for improvement was reported in psychosocial support and information about late effects.

**Conclusions:**

The naturally evolved patterns identified in the study resemble published care models: Follow-up clinics for complex needs, specialist-led care for unstable disease states, and general practitioner-led care for stable conditions.

**Supplementary Information:**

The online version contains supplementary material available at 10.1007/s00432-023-04889-7.

## Background

One out of three individuals will be affected by cancer during their lifetime (Jemal et al. 2011). Due to improvements in diagnosis and treatment, the proportion of cancer survivors is continuously increasing. At present, about half of cancer patients will survive for 10 years or more (Allemani et al. 2018; Lagergren et al. 2019). From a patient’s perspective, cancer survivorship has been divided into three phases: acute defined as cancer diagnosis and treatment; extended defined as the period following treatment; and permanent equivalent to long-term control or cure (Mullan 1985; Mayer et al. 2017).

Because cancer survivors are at increased risk for adverse physical, mental and social consequences, follow-up care is an important component of long-term support (Jacobs and Shulman 2017). How best to provide this support is a matter of debate (Jefford et al. 2022). While comparative studies have been conducted for the most common types of cancer, such as breast, prostate, gynecological, or colorectal (Jacobs and Shulman 2017), little is known about follow-up care for hematologic malignancies (Laidsaar-Powell et al. 2019). Collectively, they are the fourth most frequent type of cancer comprising 9% of all cancer diagnoses (Smith et al. 2011) and 8% of all cancer survivors (Parry et al. 2011).

Blood cancer differs from solid tumors in various ways. First, owing to the hematopoietic system’s complex structure, an unusually large number of subtypes have been defined (Khoury et al. 2022; Alaggio et al. 2022). Second, because of its disseminated nature, systemic drug- or cell-based therapies prevail over local procedures. Radiotherapy is used with decreasing frequency and surgery is not part of the therapeutic armamentarium (Izar et al. 2016; Rezvani et al. 2016; Specht 2016; Slaney et al. 2018). Third, compared to other cancer types, chemotherapy tends to be more intense with more severe and longer lasting side effects (Hodgson 2015). Fourth, the proportion of long-term survivors is high. Despite dissemination, aggressive forms of blood cancer can often be cured and indolent forms can be controlled over prolonged periods of time or may not require treatment at all.

To gather information about the follow-up care received by blood cancer survivors from the University Hospital of Essen, the oldest and one of the largest comprehensive cancer centers in Germany, and to identify and compare patterns of care, we performed a questionnaire-based observational study consisting of two parts. The ‘Aftercare in Blood Cancer Survivors’ (ABC) study included blood cancer survivors who had been diagnosed at our institution before 2010, with an interval from the last intense treatment of at least three years. The retrospective part of the study was based on information provided by the survivors. Its major goal was to identify patterns in follow-up care and characterize the institutions that provide it. In the ensuing prospective part of the study, we compared these institutions with respect to health-related outcomes and resource use.

This report summarizes recruitment of blood cancer survivors and patterns of follow-up care identified in the retrospective part of the ABC study.

## Methods

### Eligibility

Patients 18 years or older diagnosed with and/or treated for a hematologic malignancy at the University Hospital of Essen were eligible for the study, provided that the interval between study inclusion and the date of diagnosis (for untreated patients) or the end of last treatment (for primary disease relapse or a second primary malignancy) was ≥ 3 years. In patients receiving continuous oral medication or low-dose maintenance therapy after intensive induction, eligibility started 3 years after treatment initiation or end of induction, respectively. Patients exclusively treated in childhood or adolescence were not eligible. Conditions included monoclonal gammopathy of undetermined significance (MGUS), multiple myeloma (MM), indolent non-Hodgkin lymphoma including chronic lymphocytic leukemia (iNHL/CLL), myeloproliferative neoplasms including chronic myeloid leukemia (MPN/CML), myelodysplastic syndromes (MDS), aggressive non-Hodgkin or Hodgkin’s lymphoma (aNHL/HL), and acute myeloid or acute lymphoblastic leukemia (AML/ALL). Irrespective of the underlying disease, allogeneic transplant recipients were allocated to a separate group (AlloTx). Patients were categorized according to diagnostic group, treatment, follow-up institution, and year of follow-up (4–5, 6–10, > 10 years).

### Study design

The ABC study was an observational study. It was performed from October 2013 to December 2016, comprising a 6-month retrospective and an 18-month prospective part. The study was approved by the ethics committee of the University of Duisburg-Essen (no. 14-5692-BO).

In the retrospective part, eligible patients were identified by the hospital information system, tumor board reports, and discharge letters of the Department of Hematology spanning the period from 1999 to 2010. Patients were informed by mail about the purpose of the study and invited to complete a 118-item questionnaire specifically designed for the study. Eleven questions pertained to general aspects of follow-up care (see Supplementary Material). Quality of life was assessed by the German versions of the EORTC QLQ C-30 and Hospital Anxiety and Depression Scale (HADS) questionnaires. Patients not responding within 4–6 weeks were contacted by mail again, and patients failing to respond to the second invitation were reminded by phone (Stang et al. 2005).

### Statistical analysis

Frequencies are presented as numbers and compared using the chi^2^ test. Unless otherwise stated, percentages refer to the total number of patients, i.e., they are not corrected for missing data. Continuous data are presented as median, first and third quartile (interquartile range IQR). They are compared using the Kruskal–Wallis test and graphically displayed as box whisker plots, diamonds representing means. All analyses are exploratory assuming statistical significance at *p* ≤ 0.05.

The patients’ response behavior was analyzed by logistic regression with stepwise selection of variables (inclusion and exclusion). Two binary variables served as dependent variables: 1. participating patients versus eligible non-participants, and 2. patients refusing participation versus patients disregarding the invitation to participate. The independent variables included age, sex, disease group, time from diagnosis, time from last treatment, relapse, and place of residence.

The quality-of-life scales were normalized to attain maximum power. The following transformations from the log-transform family were found to yield well normalized scales suitable for analysis of variance (ANOVA) and co-variance (ANCOVA):

$$Y = A + B \times \ln \left( {C + D \times X} \right)$$

$$X = {\text{Global health scale }}\left( {{\text{EORTC QLQ C}} - 30} \right): \, A = 5, \, B = - 1, \, C = 140, \, D = - 1$$

$$X = {\text{Functional scale }}\left( {{\text{EORTC QLQ C}} - 30} \right): \, A = 5, \, B = - 1, \, C = 120, \, D = - 1$$

$$X = {\text{Symptom scale }}\left( {{\text{EORTC QLQ C}} - 30} \right): \, A = 0, \, B = 1, \, C = 20, \, D = 1$$

$$X = {\text{Anxiety scale }}\left( {{\text{HADS}}} \right): \, A = 0, \, B = 1, \, C = 10, \, D = 1$$

$$X = {\text{Depression scale }}\left( {{\text{HADS}}} \right): \, A = 0, \, B = 1, \, C = 1, \, D = 1$$

Note that the sense of direction is maintained, i.e., with increasing *X* *Y* also increases. The transformed scales and the nominal factor ‘follow-up institution’ were used to calculate general linear models adjusting for age and sex. *P* values corrected for multiple testing pertain to any group difference as well as pointwise deviation from the common least-squares mean.

## Results

### Participation of blood cancer survivors

#### Participation

**Fig. 1:**
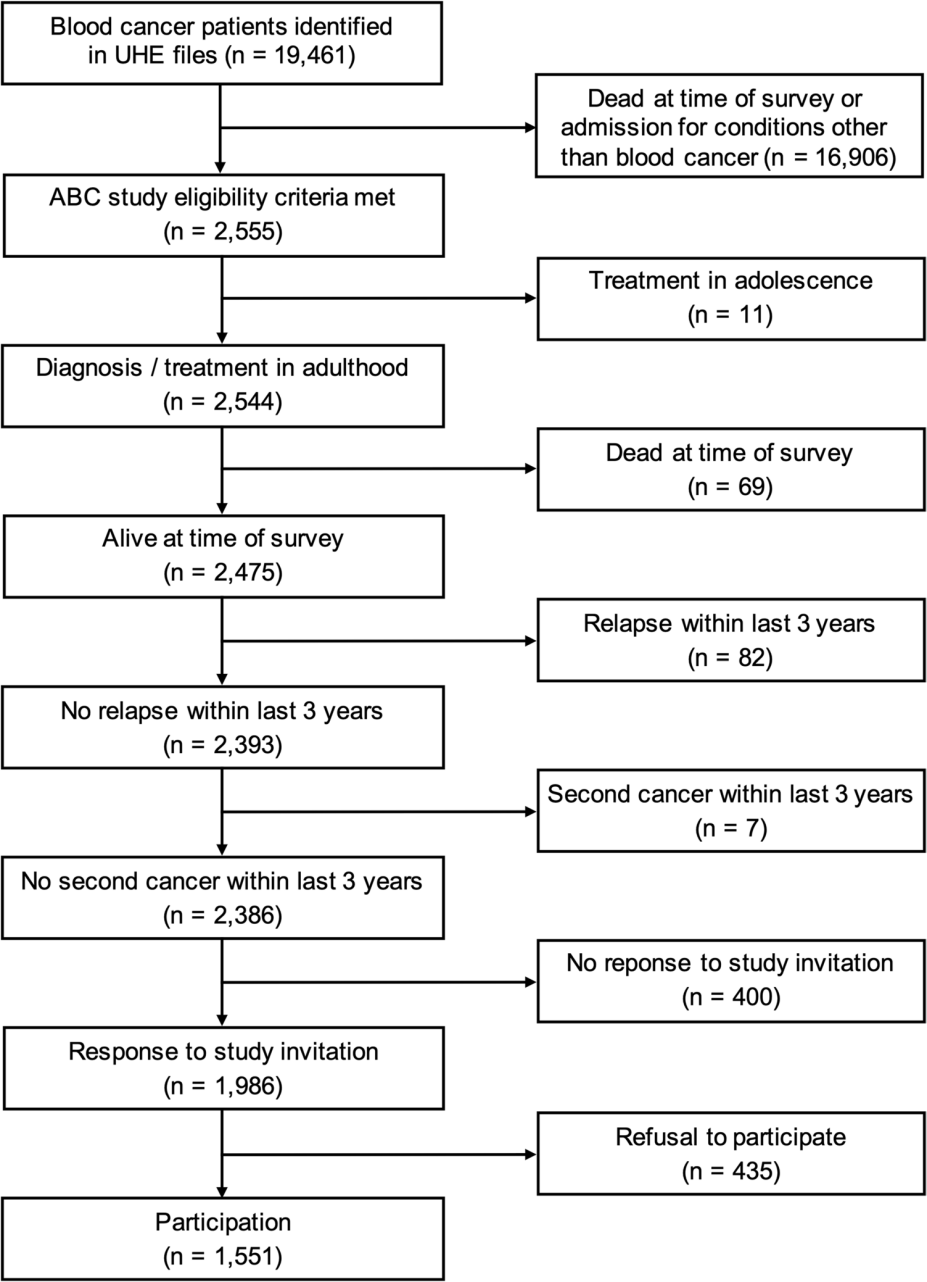
Flow diagram of long-term blood cancer survivors (from identification in the University Hospital of Essen (UHE) patient files to participation in the ABC study)

Of 19461 blood cancer patients identified in the hospital files, 2555 patients that fulfilled the eligibility criteria according to the data available at the University Hospital of Essen were asked to participate in the study. Eleven patients had to be excluded because of previously unrecognized exclusive treatment in adolescence, 69 were deceased, 82 had experienced a relapse within the last 3 years, and 7 had developed a second cancer within the last 3 years, leaving 2386 living patients meeting the inclusion criteria (Fig. 1). Of these, 1551 (65.0%) agreed, 435 (18.2%) refused, and 400 (16.8%) ignored our invitation to participate.

#### Disease groups

Nineteen participants had MGUS, 46 MM, 288 iNHL/CLL, 326 MPN/CML, 45 MDS, 514 aNHL/HL, and 313 AML/ALL. Allogeneic transplantation was performed in 554 patients (35.7%), most frequently in survivors with a history of MDS (40/45, 88.9%), AML/ALL (239/313, 76.4%), or MPN/CML (219/326, 67.2%) (Table 1). Because health issues arising 3 years or later after transplantation are more likely to be related to the procedure than the disease, AlloTx was established as an independent category, comprising all transplanted survivors irrespective of the underlying disease. The other categories were restricted to non-transplanted survivors. About half of the participants were beyond 10 years of follow-up, about a third between years 6 and 10, and a minority in years 4 and 5 (Table 1).

**Table 1:** Characteristics of study participants and eligible non-participants according to data from the University Hospital of Essen patient files

	Participants		Non-participants	
Total number of survivors	1551		835	
Age at study entry—years^a^	57.6 (23.0–91.2)		56.1 (21.9–94.3)	
Age at first diagnosis—years^a^	45.6 (15.3^b^-85.2)		44.3 (10.0–89.8)	
Time from diagnosis—years^a^	10.5 (3.0–40.7)		10.8 (3.0–39.3)	
Time from last treatment—years^ac^	8.9 (3.0–36.0)		10.1 (3.0–38.9)	
Male—number of survivors (%)^d^	841 (54.2%)		475 (56.9%)	
Female—number of survivors (%)^d^	710 (45.8%)		360 (43.1%)	
Follow-up period—number of survivors (%)^d^				
Year 4–5	292 (18.8%)		128 (15.3%)	
Year 6–10	528 (34.1%)		298 (35.7%)	
Year > 10	731 (47.1%)		409 (49.0%)	

Disease group—number of survivors	No AlloTx	AlloTx	No AlloTx	AlloTx
MGUS	19	0	14	0
MM	37	9	37	5
iNHL/CLL	264	24	129	7
MPN/CML	107	219	75	82
MDS	5	40	8	10
aNHL/HL	491	23	335	10
AML/ALL	74	239	47	76
Total	997	554	645	190

*MGUS* monoclonal gammopathy of undetermined significance, *MM* multiple myeloma, *iNHL* indolent non-Hodgkin lymphoma, *CLL* chronic lymphocytic leukemia, *MPN* myeloproliferative neoplasm, *CML* chronic myeloid leukemia, *MDS* myelodysplastic syndrome*, aNHL* aggressive non-Hodgkin lymphoma, *HL* Hodgkin lymphoma, *AML* acute myeloid leukemia, *ALL* acute lymphoblastic leukemia, *AlloTx* allogeneic blood stem cell transplantation

^a^Median (range)

^b^Patients first diagnosed before age 18 qualified for the study only if the last treatment was performed after age 18

^c^Time from last treatment in 1279 treated participants (82.5% of total participants) and 608 treated non-participants (72.8% of total non-participants)

^d^Percent of total number of survivors

#### Features of participating versus non-participating survivors

Baseline characteristics of study participants and eligible non-participants are provided in Table 1. Consent to participate was positively correlated with the interval from primary diagnosis (maximum likelihood estimate ± standard error 0.0363 ± 0.0169 per year, *p = *0.0316) and affiliation to the AlloTx (0.7258 ± 0.1213, *p < *0.0001) or iNHL/CLL groups (0.3236 ± 0.1341, *p = *0.0159). It was negatively correlated with the interval from last treatment (− 0.0609 ± 0.0183 per year, *p = *0.0009). Active refusal (versus disregard of the invitation to participate) was positively correlated with age (0.0406 ± 0.0049 per year, *p*  <  0.0001) and relapse (0.5761 ± 0.2280, *p = *0.0115), and negatively correlated with male sex (− 0.1929 ± 0.0744, *p = *0.0095).

### Providers of follow-up care

#### Follow-up physicians

The survivors named 1070 physicians involved in follow-up care. Of these, 223 were hematologists and medical oncologists (which is a single medical specialty in Germany; subsequently referred to as ‘oncologists’), 366 non-oncological internists, 386 general practitioners, and 95 represented other disciplines. The majority (921/1070, 86.1%) worked in private practice, but a substantial proportion of oncologists (101/223, 45.3%) was hospital-based.

#### Follow-up institutions

The study participants were asked to indicate their major follow-up institution. The answers revealed three groups: first, the university hospital (1045 patients, 67.4%); second, oncologists outside the university hospital (231 patients, 14.9%) working at non-university hospitals (67 patients) or in private practice (164 patients); and third, other physicians in private practice (203 patients, 13.1%) trained as non-oncological internists (99 patients), general practitioners (94 patients), or in other disciplines (10 patients), collectively referred to as ‘internists/practitioners’. Seventy-two blood cancer survivors (4.6%) did not undergo follow-up care, including 49 who had never made use of it, and 23 who had terminated it. Over time the number of patients cared for at the university hospital decreased from 78.8 to 60.5%, and the number of patients seen by internists/practitioners increased from 4.8 to 19.2% (Table 2).

**Table 2:** Follow-up institutions by time period

Institution	Number of survivors per institution/total number per time period (%)			
	Year 4–5	Year 6–10	Year > 10	All periods
University Hospital Essen	230/292 (78.8%)	373/528 (70.6%)	442/731 (60.5%)	1045/1551 (67.4%)
External oncologist	40/292 (13.7%)	92/528 (17.4%)	99/731 (13.5%)	231/1551 (14.9%)
Internist/practitioner	14/292 (4.8%)	49/528 (9.3%)	140/731 (19.2%)	203/1551 (13.1)
No follow-up care	8/292 (2.7%)	14/528 (2.7%)	50/731 (6.8%)	72/1551 (4.6)

chi^2^ test comparing the time periods year 4–5, 6–10, and > 10, *p < *0.0001

There were significant differences in the spectrum of blood cancer survivors seen by different follow-up institutions (*p*  <  0.0001). Although most survivors (allogeneic transplant recipients in particular) were followed up at the university hospital, patients with MGUS and MDS were most often seen by external oncologists who also cared for a substantial proportion of patients with multiple myeloma, myeloproliferative disorders, or indolent lymphomas (Table 3). Internists/practitioners focused on acute leukemia and lymphoma. Abstention from follow-up care was disproportionately frequent in MGUS, AML/ALL, and aNHL/HL.

**Table 3:** Follow-up institutions by disease group

Disease group	Number of survivors per institution/total number per disease group (%)			
	University hospital	External oncologist	Internist/practitioner	No follow-up care
No AlloTx				
MGUS	4/19 (21.1%)	7/19 (36.8%)	3/19 (15.8%)	5/19 (26.3%)
MM	21/37 (56.8%)	13/37 (35.1%)	2/37 (5.4%)	1/37 (2.7%)
iNHL/CLL	166/264 (62.9%)	49/264 (18.5%)	38/264 (14.4%)	11/264 (4.2%)
MPN/CML	69/107 (64.5%)	29/107 (27.1%)	8/107 (7.5%)	1/107 (0.9%)
MDS	2/5 (40.0%)	3/5 (60.0%)	0/5 (0.0%)	0/5 (0.0%)
aNHL/HL	301/491 (61.3%)	75/491 (15.3%)	80/491 (16.3%)	35/491 (7.1%)
AML/ALL	35/74 (47.3%)	11/74 (14.9%)	21/74 (28.4%)	7/74 (9.4%)
AlloTx	447/554 (80.7%)	44/554 (7.9%)	51/554 (9.2%)	12/554 (2.2%)

*AlloTx* allogeneic blood stem cell transplantation, *MGUS* monoclonal gammopathy of undetermined significance, *MM* multiple myeloma, *iNHL* indolent non-Hodgkin lymphoma, *CLL* chronic lymphocytic leukemia, *MPN* myeloproliferative neoplasm, *CML* chronic myeloid leukemia, *MDS* myelodysplastic syndrome, *aNHL* aggressive non-Hodgkin lymphoma, *HL* Hodgkin lymphoma, *AML* acute myeloid leukemia, *ALL* acute lymphoblastic leukemia, *p < *0.0001 for spectrum of patients seen at different follow-up institutions (chi^2^ test)

### Features of follow-up care

#### Follow-up intervals

1241 of 1551 responding blood cancer survivors (80.0%) reported to have regular intervals between follow-up visits, and 485 of 1442 (33.6%) were reminded of the scheduled date by their physician. The proportion of survivors with regular intervals was the highest in MDS (5/5, 100.0%) and MPN/CML (95/107, 88.8%), and the lowest in AML/ALL (51/74, 68.9%) and MGUS (11/19, 57.9%). The reported intervals became significantly longer with increasing follow-up time, with 3-month intervals prevailing in years 1 and 2, 6-month intervals in years 3 to 5, and yearly intervals thereafter (Table 4). Monthly visits were significantly more frequent in survivors with a history of allogeneic transplantation than in other survivors.

**Table 4:** Follow-up intervals as reported by blood cancer survivors

Interval	Number of survivors affected/total number responding (%)^a^			*p*
	Year 1–2	Year 3–5	Year > 5	
Monthly	433/1248 (34.7%)	106/1201 (8.8%)	53/1052 (5.0%)	< 0.0001
Three-monthly	538/1248 (43.1%)	372/1201 (31.0%)	180/1052 (17.1%)	
Six-monthly	122/1248 (9.8%)	522/1201 (43.5%)	245/1052 (23.3%)	
Yearly	50/1248 (4.0%)	131/1201 (10.9%)	441/1052 (41.9%)	
Monthly visits in relation to treatment				
No allogeneic transplantation	128/787 (16.3%)	40/759 (5.3%)	25/646 (3.7%)	0.0007
Allogeneic transplantation	305/461 (66.2%)^b^	66/442 (14.9%)^b^	28/406 (6.9%)^c^	

^a^The numbers do not add up to 100% because some survivors had irregular intervals or intervals deviating from those listed

^b^*p < *0.0001 compared to non-transplanted survivors

^c^*p = *0.0289 compared to non-transplanted survivors

*p,* chi^2^ test

#### Investigations

The survivors were asked to name the investigations performed at the follow-up visits and quantify their frequency on a 3-point scale (always, sometimes, never). During most visits, the physicians took the patient history, most consistently at the university hospital (Table 5). Special psychological support was rare. Blood was drawn by oncologists more often than by internists/practitioners. External oncologists performed a physical examination less often than university hospital oncologists and internists/practitioners, and they more often ordered computed tomography and magnetic resonance imaging.

**Table 5:** Investigations at follow-up visits as reported by blood cancer survivors

Investigation	Number of survivors affected/total number responding (%)			*p*
	University hospital	External oncologist	Internist/practitioner	
Conversation (patient history)				
Always	980/999 (98.1%)	200/222 (90.1%)	137/163 (84.1%)	< 0.0001
Sometimes	16/999 (1.6%)	21/222 (9.5%)	24/163 (14.7%)	
Never	3/999 (0.3%)	1/222 (0.4%)	2/163 (1.2%)	
Special psychological support				
Always	55/757 (7.3%)	12/166 (7.2%)	7/115 (6.1%)	0.2156
Sometimes	177/757 (23.4%)	53/166 (31.9%)	31/115 (26.9%)	
Never	525/757 (69.3%)	101/166 (60.9%)	77/115 (67.0%)	
Physical examination				
Always	662/952 (69.5%)	123/204 (60.3%)	110/152 (72.4%)	0.0012
Sometimes	272/952 (28.6%)	68/204 (33.3%)	36/152 (23.7%)	
Never	18/952 (1.9%)	13/204 (6.4%)	6/152 (3.9%)	
Blood sampling				
Always	1011/1015 (99.6%)	223/228 (97.8%)	169/186 (90.9%)	< 0.0001
Sometimes	4/1015 (0.4%)	4/228 (1.8%)	17/186 (9.1%)	
Never	0/1015 (0.0%)	1/228 (0.4%)	0/186 (0.0%)	
Bone marrow sampling				
Always	8/761 (1.1%)	3/164 (1.8%)	1/115 (0.9%)	0.7127
Sometimes	320/761 (42.0%)	68/164 (41.5%)	42/115 (36.5%)	
Never	433/761 (56.9%)	93/164 (56.7%)	72/115 (62.6%)	
Conventional radiography				
Always	124/868 (14.3%)	20/181 (11.0%)	19/122 (15.6%)	0.0489
Sometimes	497/868 (57.2%)	93/181 (51.4%)	58/122 (47.5%)	
Never	247/868 (28.5%)	68/181 (37.6%)	45/122 (36.9%)	
Ultrasonography				
Always	362/898 (40.3%)	97/206 (47.1%)	69/147 (47.0%)	< 0.0001
Sometimes	308/898 (34.3%)	89/206 (43.2%)	49/147 (33.3%)	
Never	228/898 (25.4%)	20/206 (9.7%)	29/147 (19.7%)	
Computed tomography				
Always	19/808 (2.4%)	21/186 (11.3%)	9/128 (7.0%)	< 0.0001
Sometimes	347/808 (42.9%)	94/186 (50.5%)	52/128 (40.6%)	
Never	442/808 (54.7%)	71/186 (38.2%)	67/128 (52.3%)	
Magnetic resonance imaging				
Always	13/766 (1.7%)	13/169 (7.7%)	2/117 (1.7%)	0.0001
Sometimes	198/766 (25.8%)	53/169 (31.4%)	32/117 (27.4%)	
Never	555/766 (72.5%)	103/169 (60.9%)	83/117 (70.9%)	

*p,* chi^2^ test

#### Additional help

789 blood cancer survivors (50.9%) reported that they did not require help beyond the follow-up visits. Sources of further help were family and friends for 506 (32.6%), other physicians or therapists for 321 (20.7%), religious caregivers for 80 (5.2%), and advocacy groups for 62 survivors (4.0%). There were no statistically significant differences between follow-up institutions except for help from family and friends that was the least often sought by survivors treated by internists/practitioners (Table 6). Allogeneic transplantation was associated with significantly more frequent help from family and friends (220/554 [39.7%] versus 286/997 [28.7%], *p < *0.0001), other physicians and therapists (161/554 [29.1%] versus 160/997 [16.0%], *p < *0.0001), religious caregivers (41/554 [7.4%] versus 39/997 [3.9%], *p = *0.0029), and advocacy groups (37/554 [6.7%] versus 25/997 [2.5%], *p < *0.0001) than disease management without transplantation.

**Table 6:** Help beyond follow-up consultations

Source of help	Number of survivors affected/total number per institution (%)				*p*
	University hospital	External oncologist	Internist/practitioner	No follow-up care	
Family and friends	375/1045 (35.9%)	78/231 (33.8%)	49/203 (24.1%)	4/72 (5.6%)	0.0053
Other physicians and therapists	237/1045 (22.7%)	50/231 (21.6%)	34/203 (16.7%)	0/72 (0.0%)	0.1723
Religious caregivers	65/1045 (6.2%)	9/231 (3.9%)	6/203 (3.0%)	0/72 (0.0%)	0.0923
Patient advocacy groups	49/1045 (4.7%)	10/231 (4.3%)	3/203 (1.5%)	0/72 (0.0%)	0.1121

*p,* chi^2^ test (excluding survivors without follow-up care)

#### Suggestions to improve follow-up care

The survivors were offered nine possible ways to improve follow-up care (Table 7). About half of 1262 responding survivors were content with the present situation. The most frequently suggested improvements were additional medical information (280/1262 survivors, 22.1%), support by a psychotherapist (224/1262, 17.7%), help with retirement issues (200/1262, 15.8%), and psychological support by a physician (137/1262, 10.9%). Free-text comments adding further areas of improvement were provided by 300 survivors. The most frequent suggestions were related to long-term side effects (28/300, 9.3%) and consistent allocation to the same follow-up physician (27/300, 9.0%).

**Table 7:** Suggestions to improve follow-up care

	Number of survivors affected/total number responding (%)			*p*
	University hospital	External oncologist	Internist/practitioner	
Improvements pre-specified in the questionnaire				
No improvements required	464/910 (51.0%)	98/194 (50.5%)	77/158 (48.7%)	0.8714
Psychological support by a physician	92/910 (10.1%)	17/194 (8.8%)	28/158 (17.7%)	0.0106
Psychological support by a psychotherapist	158/910 (17.4%)	32/194 (16.5%)	34/158 (21.5%)	0.3985
Supervised group psychotherapy	32/910 (3.5%)	5/194 (2.6%)	6/158 (3.8%)	0.7741
Spiritual support by a religious worker	10/910 (1.1%)	3/194 (1.5%)	4/158 (2.5%)	0.3417
Access to a patient advocacy group	34/910 (3.7%)	8/194 (4.1%)	3/158 (1.9%)	0.4655
Additional medical information	193/910 (21.2%)	52/194 (26.8%)	35/158 (22.2%)	0.2346
Help with employment issues	58/910 (6.4%)	12/194 (6.2%)	11/158 (7.0%)	0.9520
Help with retirement issues	151/910 (16.6%)	27/194 (13.9%)	22/158 (13.9%)	0.5067
Help with home care issues	38/910 (4.2%)	6/194 (3.1%)	2/158 (1.3%)	0.1782
Free-text suggestions				
Long-term side effects (information, management)	19/217 (8.8%)	6/45 (13.3%)	3/38 (7.9%)	0.5977
Consistent allocation to a single follow-up physician	24/217 (11.1%)	2/45 (4.4%)	1/38 (2.6%)	0.1258
Help with rehabilitation issues	12/217 (5.5%)	1/45 (2.2%)	0/38 (0.0%)	0.2283
Information about alternative medicine	6/217 (2.8%)	2/45 (4.4%)	0/38 (0.0%)	0.4450
Reminder of the scheduled visit	4/217 (1.8%)	3/45 (6.7%)	0/38 (0.0%)	0.0887

*p,* chi^2^ test

#### Satisfaction with follow-up care

**Fig. 2:**
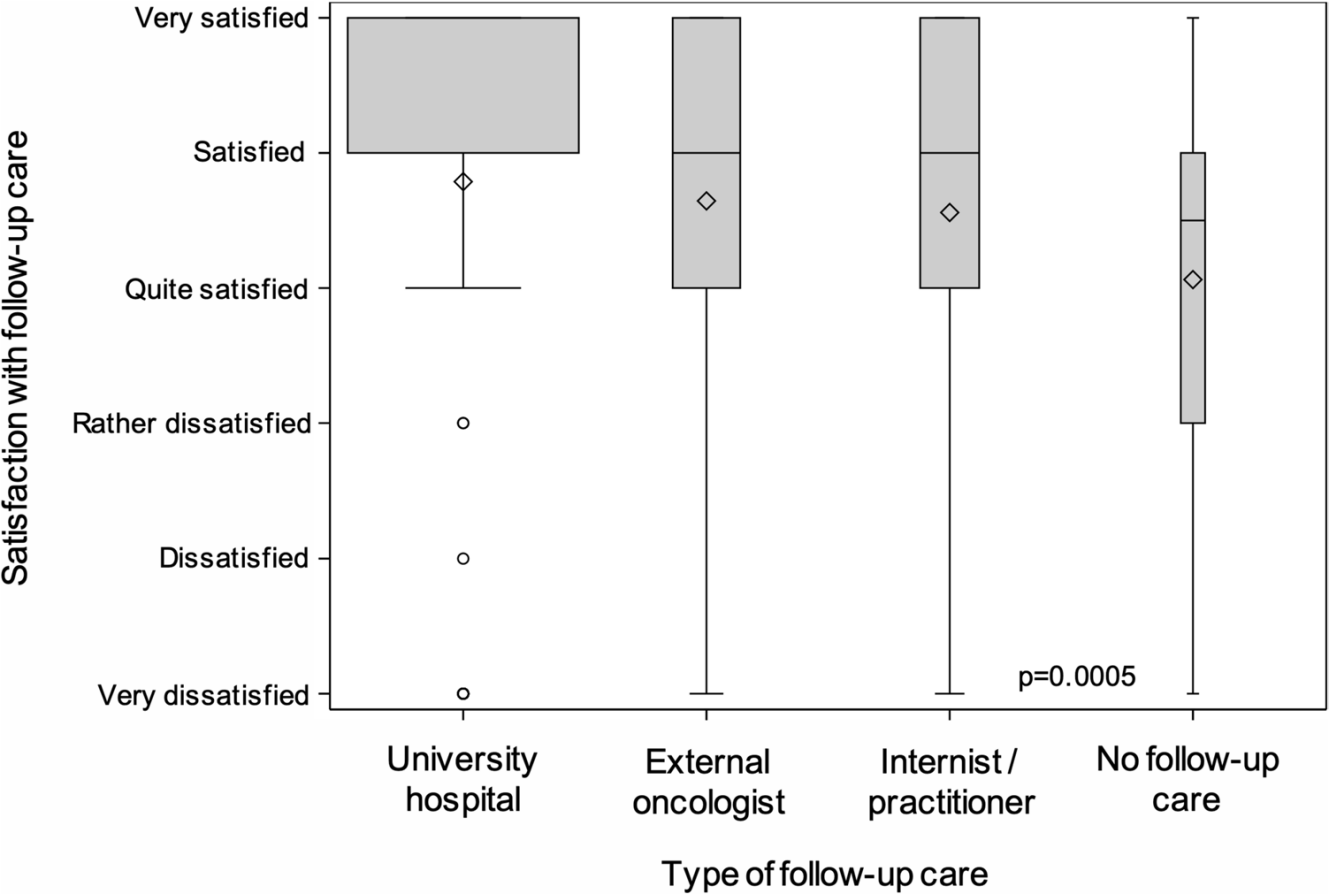
Satisfaction of blood cancer survivors with follow-up care delivered by different follow-up institutions. University hospital 1031 of 1045 survivors responding; external oncologist 228/231; internist/practitioner 186/203; no follow-up care 16/72 (the width of the boxes corresponds to the number of survivors responding)

The study participants were asked to grade their satisfaction with the follow-up care received using a 6-point scale. Survivors followed up at the university hospital had the highest and survivors not undergoing follow-up care had the lowest level of satisfaction, but the number of responses received from the latter was small (Fig. 2). Statistically significant differences between blood cancer survivors with or without a history of allogeneic transplantation or survivors in different periods of follow-up care were not observed (data not shown).

### Quality of life

**Fig. 3:**
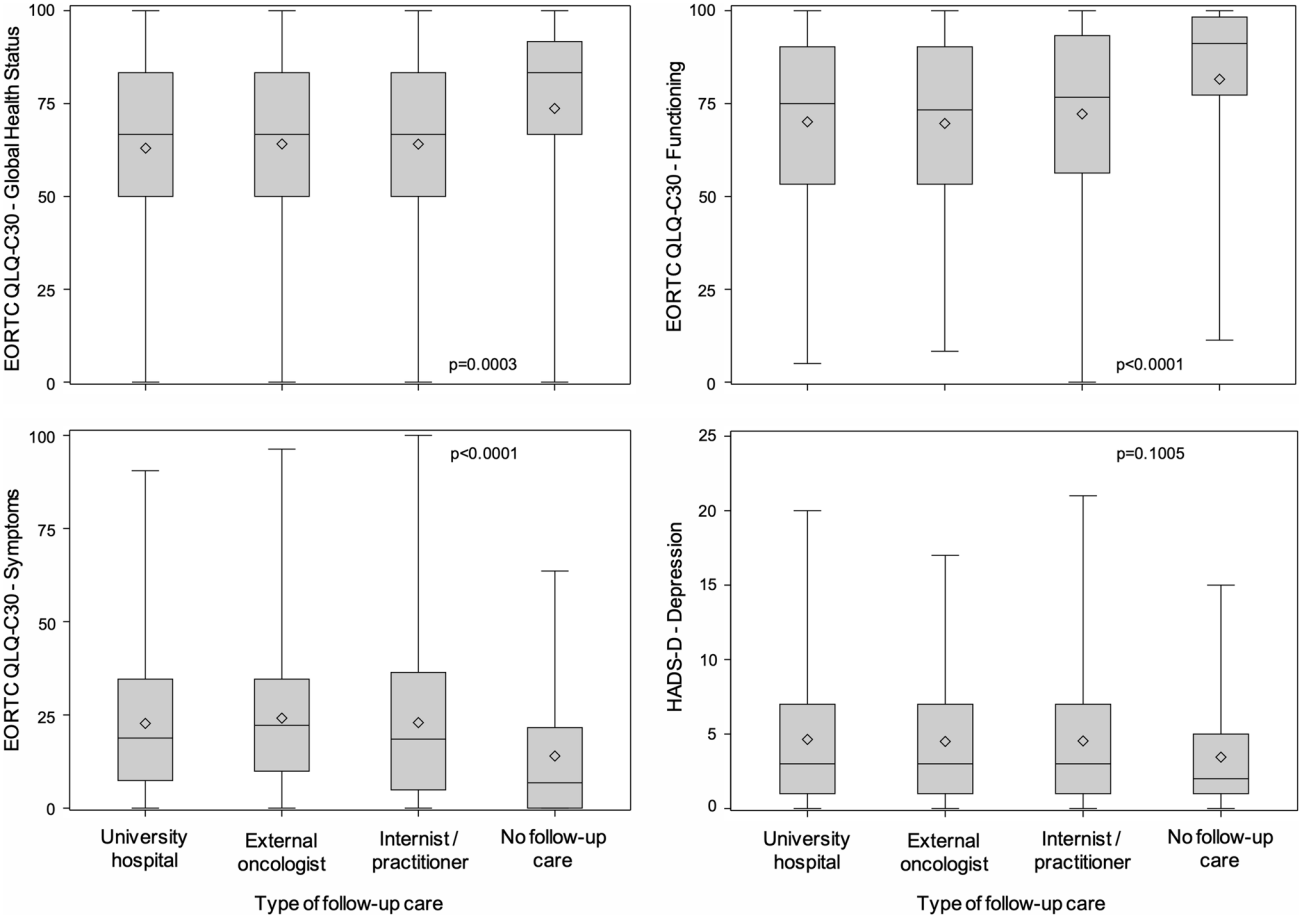
Quality of life in long-term blood cancer survivors by follow-up institution as assessed by the EORTC QLQ C-30 (global health status 1519 responding survivors; functioning 1521; symptoms 1520) and HADS questionnaires (depression 1459). The results of the HADS anxiety scale (not shown) were similar to those of the depression scale. University hospital 979–1023 survivors responding; external oncologist 219–231; internist/practitioner 192–197; no follow-up care 69–70. The p values are adjusted for age and sex and corrected for multiple testing

The assessment was restricted to the broad domains ‘global health’, ‘functioning’ (physical, role, cognitive, emotional, and social combined), and ‘symptoms’ of the EORTC QLQ C-30 questionnaire, and ‘anxiety’ and ‘depression’ of the HADS questionnaire. Quality of life was the best in survivors not undergoing follow-up care. There were no significant differences between follow-up institutions (Fig. 3).

## Discussion

The most important findings from the retrospective part of the ABC study are the following: first, the survivors’ willingness to engage in the study was high; second, almost all participants received follow-up care; and third, there were significant differences between follow-up institutions.

Using a ‘repeated reminding’ approach successfully employed before (Stang et al. 2005), almost two-third of eligible patients consented to participate, which is within the range of other large questionnaire-based blood cancer follow-up studies (participation rate 37–78%) (Oerlemans et al. 2012; Parry et al. 2012; Hall et al. 2014; Korszun et al. 2014; Pophali et al. 2020; Armenian et al. 2022; Bhatia et al. 2023). Participation was particularly high in survivors with a long interval from primary diagnosis. Almost half of the participants were beyond year 10 of follow-up care. Thus, the ABC study contributes to filling the knowledge gap in late periods of cancer survivorship (Gallicchio et al. 2021).

More than 95% of blood cancer survivors received follow-up care. This is unlikely to represent the real-world situation. The study design did not allow us to quantify follow-up care utilization among survivors not participating in the study. However, even if none of the non-participants received follow-up care, its overall use would still exceed 60%. As expected, most participants had been diagnosed with curable types of blood cancer (aNHL/HL and AML/ALL), had received curative treatments (AlloTx), or suffered from incurable diseases with good prognosis (MGUS, iNHL/CLL, and MPN/CML). Prognostically unfavorable diseases such as MM or MDS (Khoury et al. 2022; Alaggio et al. 2022) were underrepresented.

Even after years or decades, more than two-third of survivors still came to the university hospital for follow-up care. This may in part be explained by the fact that the transfer of patients to the follow-up program at their own institution is a reflex action for many university hospital physicians. In addition, both patients and oncologists tend to have little confidence in general practitioners to provide follow-up care (Potosky et al. 2011; Jefford et al. 2022).

The large number of patients taking part in the ABC study allowed us to identify two other important care providers. About one-sixth of the survivors were seen by non-university oncologists, and the same percentage by non-oncological internists/practitioners. Most of these worked in private practice. The disease spectrum differed at the three institutions. Patients with a history of allogeneic transplantation were overrepresented at the university hospital. Patients with incurable diseases requiring continuous monitoring (MDS, MGUS, MM, MPN/CML, and iNHL/CLL) were disproportionately often seen by non-university oncologists. By contrast, survivors with curable diseases in stable remission (AML/ALL and aNHL/HL) were frequently followed up by internists/practitioners. Although the patterns of follow-up care observed in the ABC study evolved naturally, they resemble published care models: long-term follow-up clinics (e.g., university hospitals) for survivors with complex needs and a high risk of late effects, specialist-led care for unstable disease states, and general practitioner-led care for stable conditions (Jefford et al. 2022). With increasing follow-up time, an increasing proportion of survivors moved to internist/practitioner-led care. This has also been observed for solid tumor survivors (Pollack et al. 2009), possibly reflecting the assurance that the disease is cured (Oeffinger and McCabe 2006).

In the majority of survivors, follow-up intervals were described as regular, with increasing intervals corresponding to increasing time from treatment. In essence, they reflected expert opinion-based published recommendations (Majhail et al. 2012; Cheson et al. 2014). The follow-up visits were dominated by conversations, physical examination, and blood sampling. While ultrasonography was the most common imaging method in all three follow-up institutions, computed tomography and magnetic resonance imaging were strikingly overrepresented outside the university hospital. Apart from differences in the disease spectrum, dissimilar work environments may explain this observation. Physicians working in a large institution surrounded by specialists from diverse disciplines may tolerate diagnostic uncertainty better than physicians working in private practice, who may try to attain certainty using the most sensitive methods (Simpkin and Schwartzstein 2016).

About half of the survivors were completely satisfied with the follow-up care received. Better psychological support, more medical information, better management of late effects, and help with work-related issues were identified as fields requiring improvement. Similar unmet needs were reported in a systematic review of 18 previous follow-up studies in patients with hematologic malignancies (Swash et al. 2014). Psychological support for cancer patients was in its infancy when the majority of ABC study participants were diagnosed. Meanwhile, its importance has been well recognized and psychosocial care ranks among the top priorities to improve follow-up care in Europe and North America (Lagergren et al. 2019; Gallicchio et al. 2021). The same is true for late effects, whose spectrum will change with changing treatments. Expert knowledge about side effects of novel treatments may argue in favor of follow-up care by cancer specialists (Jacobs and Shulman 2017; Jefford et al. 2022).

High levels of satisfaction with follow-up care have also been reported from the Netherlands (67% of 1135 survivors with a history of a hematologic malignancy [Oerlemans et al. 2012]) and Australia (75% of 696 survivors [Hall et al. 2014]; 49% of 311 survivors [Boyes et al. 2015]). In a study from Colorado restricted to the first 4 years of follow-up care, blood cancer survivors appeared to be less satisfied, with at least one in four reporting considerable unmet needs (Parry et al. 2012). Similar to the ABC study, dissatisfaction was mostly related to deficits in psychological support and information about the disease in general and late treatment effects in particular. In some studies, physical aspects of daily living (Hall et al. 2014; Boyes et al. 2015), sexuality, and financial or employment issues also ranked high (Parry et al. 2012). Comparisons must be done with caution. While the ABC study started in year 4 of follow-up care, the other studies included the transition period immediately after chemotherapy, with no or only a minority of survivors receiving care beyond year 10.

In addition to follow-up visits, a substantial proportion of survivors sought help from family, friends, and other sources. This was particularly evident in bone marrow transplant recipients. Surprisingly, less than 5% had contacts with advocacy groups, and even fewer thought that access to such groups would improve care. This is at variance with a study from California where blood cancer survivors were more likely to make use of support groups than other cancer survivors (41% versus 1–25%). In that study, participation in group activities was perceived as a benefit by most blood cancer survivors (Owen et al. 2007). The discrepant results may be related to differences in sociocultural factors, availability of and information about support groups, patient selection, and sample size.

Not unexpectedly, quality of life was the best among patients not undergoing follow-up care. Such patients are likely a selection of favorable cases such as survivors with MGUS or a history of curable diseases, such as leukemia or lymphoma. At least with regard to the broad categories investigated, quality of life did not differ among follow-up institutions. Our study did not include a non-cancer control group matched for other factors. Therefore, we were unable to compare the blood cancer survivors with the general population.

Limitations of the ABC study’s retrospective part are its reliance on patient reporting, which is subject to participation and recall biases, and the exclusion of patients who died before the study was conducted (prevalence-incidence bias). Patients alive at the time of the survey are a selection of individuals with a favorable disease course. Whether the information provided by them is representative of the entire population remains uncertain. A further limitation is the fact that any comparison of institutions is confounded by differences in disease spectrum and follow-up duration. A strength of our study is its large size with a high proportion of participants in late periods of follow-up care.

## Conclusions

Almost all blood cancer survivors participating in the ABC study received follow-up care. There were three major follow-up institutions that exhibited significant differences with respect to disease groups and diagnostic procedures. The naturally evolved patterns observed in the study resemble published care models: follow-up clinics for complex needs, specialist-led care for unstable disease states, and general practitioner-led care for stable conditions. Irrespective of institution, the survivors’ satisfaction with follow-up care was high, but there was room for improvement in psychosocial support and information about late effects.

## Supplementary Information

Below is the link to the electronic supplementary material.

Supplementary file1 (PDF 181 KB)

## Data Availability

The datasets used and analyzed during this study are available from the corresponding author on reasonable request.
