# Supplementary material for: Patient-reported patterns of follow-up care in the ‘Aftercare in Blood Cancer Survivors’ (ABC) study
Source: J Cancer Res Clin Oncol. 2023 Jun 8;149(12):10531–42. doi: 10.1007/s00432-023-04889-7 (PMC10423115; doi:10.1007/s00432-023-04889-7)
Supplement: Supplementary file 1 — Supplementary file1 (PDF 181 KB) [file 432_2023_4889_MOESM1_ESM.pdf]

Aftercare in blood cancer survivors (ABC study)

Retrospective part – Patient questionnaire

Questions related to blood cancer follow-up care

1. **Nehmen Sie an Nachsorgeuntersuchungen für Ihre Blutkrebserkrankung teil?**

☐1 Ja, regelmäßig
☐2 Ja, unregelmäßig
☐3 Früher ja, aber jetzt nicht mehr, da die Erkrankung schon lange zurückliegt
☐4 Nein ☞ weiter mit Frage 16

2. **Wer führt die Nachsorge hauptsächlich durch?**
(Hier ist nur eine Antwort möglich!)

☐1 Meine Hausärztin, mein Hausarzt
☐2 Meine niedergelassene Hämatologin oder Onkologin / mein niedergelassener Hämatologe oder Onkologe
☐3 Eine Ärztin / ein Arzt im Krankenhaus
☐4 Jemand anderes,
und zwar: (bitte angeben): ____

3. **Erinnert Sie jemand an Ihre Nachsorgetermine?**

☐1 Nein
☐2 Ja, meine nachsorgende Ärztin / mein nachsorgender Arzt
☐3 Ja, jemand anderes,
und zwar (bitte angeben): ____

4. **Bitte nennen Sie uns ein oder zwei Ärztinnen oder Ärzte, die an der Nachsorge Ihrer Erkrankung und der allgemeinen Gesundheitsvorsorge beteiligt sind**.

Fachrichtungen können z. B. Hausärzte, Internisten, Hämatologen, Onkologen oder auch andere Disziplinen sein. Wenn Ihre Nachsorge am Universitätsklinikum Essen durchgeführt wird, nennen Sie uns bitte diejenige Ärztin oder denjenigen Arzt, der dort zuletzt für Sie zuständig war. Bitte schreiben Sie in Druckbuchstaben.

Name 1 ____

Anschrift 1 ____

Fachrichtung 1 ____
(soweit Ihnen bekannt)

Name 2 ____

Anschrift 2 ____

Fachrichtung 2 ____
(soweit Ihnen bekannt)

5. **Seit wie vielen Jahren befinden Sie sich in der Nachsorge?**

Als Beginn der Nachsorge gilt entweder das Ende der zuletzt durchgeführten intensiven Chemotherapie oder Transplantation (bei Erkrankungen, die einer intensiven Behandlung bedürfen) oder die erstmalige Feststellung der Erkrankung (bei bisher unbehandelten Störungen oder Erkrankungen, die von vornherein dauerhaft mit Tabletten behandelt wurden).

☐1 3 – 5 Jahre
☐2 6 – 10 Jahre
☐3 11 – 15 Jahre
☐4 Mehr als 15 Jahre

6. **In welchen ungefähren zeitlichen Abständen finden bzw. fanden die Nachsorgeuntersuchungen statt?**

Wenn die Nachsorge bereits mehr als 5 Jahre läuft, bearbeiten Sie bitte alle drei Spalten. Wenn sie weniger als 5 Jahre läuft, beantworten Sie bitte nur die Spalten für die Jahre 1 - 2 und die Jahre 3 - 5, und wenn sie weniger als 2 Jahre läuft, nur die Spalte für die Jahre 1 - 2.

	Jahr 1 - 2*	Jahr 3 - 5*	mehr als 5 Jahre*
Monatlich	☐1	☐1	☐1
Alle 3 Monate	☐2	☐2	☐2
Alle 6 Monate	☐3	☐3	☐3
Jährlich	☐4	☐4	☐4
Ganz unregelmäßig	☐5	☐5	☐5
Andere Zeitabstände (bitte ankreuzen und eintragen)	☐6	☐6	☐6

* Jahre nach Abschluss der Behandlung (bei intensiv behandlungsbedürftigen Erkrankungen) bzw. nach erstmaliger Feststellung der Blutkrebserkrankung (bei nicht behandlungsbedürftigen oder dauerhaft mit Tabletten behandlungsbedürftigen Erkrankungen)

7. **Bitte geben Sie an, wie oft die folgenden Untersuchungen bei den bisherigen Nachsorgeuntersuchungen durchgeführt wurden.**

	Immer	Manchmal	Nie
Allgemeines ärztliches Gespräch	☐1	☐2	☐3
Spezielle seelische Unterstützung	☐1	☐2	☐3
Körperliche Untersuchung	☐1	☐2	☐3
Blutentnahme	☐1	☐2	☐3
Knochenmarkpunktion	☐1	☐2	☐3
Röntgen	☐1	☐2	☐3
Ultraschall (Sonographie)	☐1	☐2	☐3
Computertomographie (CT)	☐1	☐2	☐3
Kernspintomographie (MRT)	☐1	☐2	☐3
Positronenemissionstomographie (PET oder PET / CT)	☐1	☐2	☐3

8. **Haben Sie über die routinemäßigen Nachsorgetermine hinaus in den vergangenen Jahren weitere Unterstützung gesucht?**
(Hier sind mehrere Antworten möglich!)

☐1 Ja, von Angehörigen und Freunden
☐2 Ja, professionelle Hilfe bei anderen Ärzten oder Therapeuten
☐3 Ja, seelsorgerische Unterstützung
☐4 Ja, in einer Selbsthilfegruppe
☐5 Ja, von einer anderen Stelle,
und zwar (bitte angeben): ____
☐6 Nein, ich habe keine weitere Unterstützung gesucht

9. **Sind Sie mit der Nachsorge Ihrer Blutkrebserkrankung zufrieden?**

☐1 Sehr unzufrieden
☐2 Unzufrieden
☐3 Eher unzufrieden
☐4 Eher zufrieden
☐5 Zufrieden
☐6 Sehr zufrieden

10. **Welche Form von Hilfe würden Sie sich zusätzlich zu der vorhandenen Betreuung besonders wünschen?**
(Hier sind mehrere Antworten möglich!)

☐01 Besprechung seelischer Probleme mit einer Ärztin / einem Arzt
☐02 Besprechung seelischer Probleme mit einer Psychotherapeutin / einem Psychotherapeuten
☐03 Besprechung seelischer Probleme mit einer Seelsorgerin / einem Seelsorger
☐04 Besprechung seelischer Probleme unter ärztlicher oder psychologischer Leitung in einer Patientengruppe
☐05 Teilnahme an einer Selbsthilfegruppe
☐06 Zusätzliche Sachinformationen über Behandlungsmöglichkeiten und Behandlungsfolgen
☐07 Unterstützung bei Arbeitsplatzwechsel oder Umschulung
☐08 Beratung in Rentenfragen
☐09 Pflegerische Betreuung zu Hause
☐10 Ich wünsche mir zusätzlich zu der vorhandenen Betreuung keine besondere Hilfe
☐11 Sonstiges,
und zwar (bitte angeben): ____

11. **Haben Sie Veränderungsvorschläge bezüglich der Nachsorge? Wenn ja, führen Sie Ihre Vorschläge bitte stichwortartig auf.**

____

____

____
